# Immunogenicity of Pigeon Circovirus Recombinant Capsid Protein in Pigeons

**DOI:** 10.3390/v10110596

**Published:** 2018-10-31

**Authors:** Tomasz Stenzel, Daria Dziewulska, Bartłomiej Tykałowski, Marcin Śmiałek, Joanna Kowalczyk, Andrzej Koncicki

**Affiliations:** Department of Poultry Diseases, Faculty of Veterinary Medicine, University of Warmia and Mazury in Olsztyn, ul. Oczapowskiego 13, 10-719 Olsztyn, Poland; daria.pestka@uwm.edu.pl (D.D.); bartlomiej.tykalowski@uwm.edu.pl (B.T.); marcin.smialek@uwm.edu.pl (M.Ś.); welencasia@gmail.com (J.K.); koncicki@uwm.edu.pl (A.K.)

**Keywords:** flow cytometry, IFN-γ gene expression, ELISA, ELISPOT, pigeon circovirus, recombinant capsid protein, vaccination

## Abstract

Pigeon circovirus (PiCV) is the most frequently diagnosed virus in pigeons and is thought to be one of the causative factors of a complex disease called the young pigeon disease syndrome (YPDS). The development of a vaccine against this virus could be a strategy for YPDS control. Since laboratory culture of PiCV is impossible, its recombinant capsid protein (rCP) can be considered as a potential antigen candidate in sub-unit vaccines. The aim of this basic research was to evaluate the immune response of pigeons to PiCV rCP. Sixty six-week-old carrier pigeons were divided into two groups (experimental immunized with PiCV rCP mixed with an adjuvant, and control immunized with an adjuvant only), and immunized twice in a 21-day interval. On the day of immunization and on two, 23, 39, and 46 days post first immunization (dpv), samples of blood, spleen, and bursa of Fabricius were collected from six birds from each group to examine anti-PiCV rCP IgY, anti-PiCV rCP IgY-secreting B cells (SBC), IFN-γ gene expression, and percentage of T CD3^+^, CD4^+^, CD8^+^, and B IgM^+^ lymphocytes. The results indicated a correct immune response to PiCV rCP both in humoral and cell-mediated immunity, which was manifested by seroconversion since 23 dpv, by a significantly higher anti-PiCV rCP IgY-SBC number on two and 23 dpv, and significantly higher IFN-γ gene expression since two dpv. There were no significant differences or trends noted between particular T and B lymphocyte subpopulations. To conclude, PiCV rCP may be deemed immunogenic and could be considered as an antigen candidate in sub-unit vaccines against PiCV infections in pigeons.

## 1. Introduction

Belonging to the genus *Circovirus* and the family *Circoviridae*, the pigeon circovirus (PiCV) [1] is thought to be one of the causative factors of the greatest health problem in global pigeon breeding: young pigeon disease syndrome (YPDS) [2]. PiCV infections are spread worldwide due to the specificity of pigeon breeding [3] like, e.g., inability to follow biosecurity procedures, especially in the case of racing birds. These birds usually take part in pigeon races or shows where hundreds or thousands of birds originating from different geographic areas have direct or indirect (watering during transport) contact with each other. For this reason, the spreading of infectious diseases in pigeons is rapid and they are affected by numerous pathogens [2,4]. The prevalence of PiCV is determined by age and health status, and affects about 70% of the global pigeon population on average [5,6,7]. Asymptomatic infections with this virus are quite common and account for 36–53% of the population [8,9,10].

The pigeon circovirus is transmitted mainly horizontally, but vertical transmission is also possible [11,12,13]. In infected pigeons, PiCV targets the bursa of Fabricius and other organs of the immune system, including the thymus and spleen [14]. The immune organs are not the only ones that are infected with PiCV. The virus or its genetic material has been detected in the liver, kidneys, intestines, brain, and skin, and recently even in the gizzard, third eyelid, and thyroid gland [4,15,16]. The main consequence of PiCV infection is the atrophy of immune system organs. Pigeon circovirus infection is also claimed to lead to the apoptosis of lymphocytes [17]. For the above reasons, PiCV is regarded as a putative immunosuppressive agent in pigeons [14,17,18]. PiCV infections could predispose birds to accompanying infections with other viruses or bacteria [2,7,19]. The clinical presentation of YPDS varies, because a combination of conditionally pathogenic microorganisms is required for disease manifestation. The type and intensity of clinical symptoms are correlated with the type of confounding factor. In pigs, infections caused by another immunosuppresive virus from the *Circoviridae* family—porcine circovirus type 2 (PCV2), leading to lymphoid atrophy, both lymphopenia and suboptimal antibody responses—were noted. The main pathogenicity of this virus has been demonstrated to result from its influence on both positive and negative selection of maturing T cells in the thymus. The PCV2 infection in pigs leads to thymocyte selection dysregulation in infected animals [20]. The above facts make YPDS similar to a swine disease caused by PCV2 called PCV2-systemic disease (PCV2-SD) [20,21]. 

The organization of the pigeon circovirus genome is typical of the virus family *Circoviridae* and consists of single-stranded spherical DNA (ss-DNA) of approximately 2030 base pairs (bp) [22]. The PiCV genome is larger than PCV2 genome (approximately 1770 base pairs) and is smaller in size than the chicken anemia virus (CAV) genome (approximately 2250 base pairs), which is the only representative of the genus *Gyrovirus* [23]. Due to the numerous differences in genome organization between circoviruses and CAV, the genus *Gyrovirus* was moved to the *Annelloviridae* family [24]. Like other circoviruses, PiCV is characterized by high genetic diversity, and based on the analysis of the full genome sequences, its five subgenotypes have been identified [9]. The genome of PiCV has an ambisense organization, with two major open reading frames (ORFs): ORF V1, which is located on the virion sense strand and encodes replicase (Rep protein), and ORF C1, which is located on the complementary sense strand and encodes the viral capsid protein (Cap protein, CP) [1,22]. Apart from the fact that CP is the main constituent of circoviral capsids, it plays an intermediate role in viral DNA penetration into the cell nucleus of the host. Investigations on psittacine circoviruses have demonstrated that the arginine-rich N-terminus of this protein has the capability to bind with host DNA using a nuclear localization sequence (NLS), which enables viral DNA penetration into a cell nucleus of the host through a complex of nuclear pores [25]. Capsids of circoviruses form repeating sub-units of CP, owing to which CP is believed to be the main antigenic protein of these viruses [26]. Immune responses induced by contact with a circovirus capsid were demonstrated in PCV2 and psittacine circovirus (psittacine beak and feather disease virus, PBFDV) [26,27]. Studies conducted on PCV2 have demonstrated the virus capsid protein to be responsible for the immune response, including the production of specific antibodies and interferon gamma (IFN-γ). For instance, the immune response in the form of appearing antibodies occurred between week two to week four after piglets’ exposure to PCV2 or to porcine circovirus capsid protein [27].

Traditional vaccines are mainly composed of live, attenuated, or inactivated pathogens; however, no laboratory protocol has so far been developed for culturing PiCV, and for this reason, the specific immunoprophylaxis of PiCV infections in pigeons is impossible [28]. Various methods for antigen production for diagnostic tests—PiCV recombinant capsid protein (rCP)—have been developed in recent years [10,28,29,30]. PiCV rCP could also be a potential candidate as a vaccine antigen, but its immunogenicity has not been confirmed in scientific research. The sub-unit vaccines based on PCV2 recombinant capsid proteins are successfully used in the prevention of PCV2-SD [31,32,33,34]. By analogy with the above, the development of a sub-unit vaccine against PiCV could protect pigeons against infections with this virus and, possibly, lower the prevalence of YPDS. 

The aim of the presented basic research was to verify the hypothesis that the pigeon circovirus recombinant capsid protein is immunogenic to pigeons. To achieve this goal, both the cell-mediated and humoral immune responses were evaluated after pigeon immunization with PiCV rCP. 

## 2. Materials and Methods 

### 2.1. Ethical Statement

The production of monoclonal antibodies in mice was carried out in observance of the I Local Ethics Committee in Wrocław (Authorization No. 70/2015), whereas experiment trial with pigeons was carried out in strict observance of the Local Ethics Committee on Animal Experimentation of the University of Warmia and Mazury in Olsztyn (Authorization No. 64/2014). The researchers made every effort to minimize the suffering of birds.

### 2.2. Pigeons

As SPF (specific pathogen free) pigeons are unavailable, sixty six-week-old carrier pigeons were obtained from a private breeder. The flock in the breeding facility is under professional veterinary service and there has been no YPDS history in this flock since 2010. Before the experiment, cloacal swabs and blood samples were collected from all birds to rule out PiCV infection with the use of the qPCR method described previously [35] and to determine the presence of antibodies against PiCV using the in-house ELISA method developed in our previous study [10]. All pigeons were also screened by bacteriological, mycological, and parasitological examinations, and no pathogenic microorganisms were found. The birds were housed in isolated units in a PCL3 biosafety facility of the Department of Poultry Diseases, Faculty of Veterinary Medicine of the University of Warmia and Mazury in Olsztyn. Every group of pigeons was housed in a separate unit. The birds were administered seed mixtures and water ad libitum throughout the experiment.

### 2.3. Experimental Design

Pigeons were divided into two groups (experimental-E and control-C) of 30 birds each. After a two-week adaptation period, pigeons from group E were immunized with PiCV rCP mixed with an adjuvant, whereas birds from group C were immunized with adjuvant only. The birds were boosted at 21 days after first immunization (35th day of the experiment). On the day of the first immunization and on day two, 23, 39, and 46 post first immunization (dpv), blood samples were collected from six birds from each group. Next, those six pigeons were euthanized and samples of their spleen and bursa of Fabricius were collected during an anatomopathological examination. Blood samples were used for the isolation of mononuclear cells for the flow cytometry (percentage of T CD3^+^, CD4^+^, CD8^+^, and B IgM^+^ lymphocytes), for qPCR for IFN-γ gene expression analysis, and to obtain sera for the determination of anti-PiCV rCP antibodies with in-house ELISA. Mononuclear cells isolated from the spleen were divided into three parts: One part was used to determine the percentage of T CD3^+^, CD4^+^, CD8^+^ and IgM^+^ B cells in a flow cytometric analysis, the second part was used for the determination of anti-PiCV rCP IgY-secreting B cells (SBC) with ELISPOT, and the third part was used in RNA extraction to evaluate the expression of gene which encodes IFN-γ. The mononuclear cells isolated from the bursa of Fabricius samples were used to determine the percentage of T CD3^+^ and B IgM^+^ cells with the flow cytometry. The experimental design is presented in Table S1.

### 2.4. PiCV Recombinant Capsid Protein (PiCV rCP)

The synthetic construct of a capsid protein gene of the Polish pigeon circovirus PL53 strain (GenBank Acc. No.: KF738860.1) optimized for the expression in bacterial systems by the GenScript company (Piscataway, NJ, USA) was cloned into the pET30a vector (Novagen, Merck-Millipore, Burlington, MA, USA). The recombinant plasmid was used for the transformation of *Escherichia* (*E.*) *coli* strain BL21 Star (DE3) (Thermo Scientific, Waltham, MA, USA) chemically competent cells using the heat shock method. The procedure of expression and protein purification was performed in accordance with the method described in our previous study [10]. To increase the solubility and similarity of the PiCV rCP to the native form, 0.3% SDS (Sigma Aldrich, Schnelldorf, Germany) was added to the target protein and it was refolded by dialyzing into a buffer (pH 8.0) composed of 50 mM Tris-HCl (Amresco, Solon, OH, USA), 0.3% SDS (Sigma Aldrich, Schnelldorf, Germany) with the volume ratio of 1:100. The dialysis was performed in 14 kDa cut-off dialysis membrane (Viskase, Willowbrook, IL, USA) for four hours and with a changed fresh buffer for an additional 16 hours with the same volume ratio. After dialysis, the sample was centrifuged at 10,000× *g* for 30 min and filtered through a 0.22 μm filter (Millex GP filter unit; Merck Millipore, Burlington, MA, USA). To avoid multiple freeze-thaws, the whole obtained protein was divided into aliquots necessary for all further procedures (pigeons’ immunization, ELISA, ELISPOT) and stored at −80 °C. Each protein aliquot was slowly defrosted on ice before use. 

### 2.5. Preparation of PiCV rCP for Immunization

The immunization dose was 20 µg of PiCV rCP per pigeon. Each dose of the immunization mixture was prepared individually by mixing a volume of protein equivalent for the immunization dose with PBS (phosphate-buffered saline) (Sigma Aldrich, Schnelldorf, Germany) up to the volume of 200 µL. This solution was mixed with 200 µL of oil-based adjuvant (Montanide ISA71 R VG) provided by Seppic (Paris, France). PBS (200 µL), mixed with an equal volume of the adjuvant served as an immunization control.

### 2.6. Determination of Anti-PiCV rCP IgY with In-House ELISA

The assay was performed on Nunc-Immuno Module plates (Thermo Scientific, Waltham, MA, USA) using the Antibody Pair Buffer Kit (Invitrogen, Carlsbad, CA, USA) according to the protocol described in our previous study [10]. Each well was coated with 100 µL of PiCV rCP (concentration 20 µg/ mL) suspended in a coating buffer B. Sera obtained from the blood of the examined pigeons were diluted (1:400) in the assay buffer, and 50 µL of the serum was deposited in each well. The rabbit anti-pigeon IgG (Antibodies-online, Atlanta, GA, USA) diluted (1:30,000) in the assay buffer were used as primary antibodies, and diluted (1:1,000) goat anti-rabbit antibodies with horseradish peroxidase (HRP) (BD Biosciences, Franklin Lakes, NJ, USA) were used as secondary antibodies. The rinsing after each step was performed with an ELx 405 automatic washer (Biotek, Winooski, VT, USA), whereas successive components were added to the samples with the use of an epMotion 5075 LH automatic pipetting station (Eppendorf, Hamburg, Germany). Optical density was measured with an ELx 800 spectrophotometer (Biotek, Winooski, VT, USA) at the wavelength of 450 nm. Data were expressed as mean OD_450_ ± standard deviation in each group for each day of sampling.

### 2.7. Isolation of Mononuclear Cells for ELISPOT, Flow Cytometry and qPCR

Mononuclear cells were isolated from the peripheral blood, spleen and bursa of Fabricius samples using the following protocol. Peripheral blood samples (1.5 mL) collected from each bird were diluted (1:1) with PBS containing 1% Fetal Bovine Serum (FBS) (Sigma Aldrich, Schnelldorf, Germany). Whole spleens were homogenized using a manual Dounce tissue grinder (Kimble, DWK Life Sciences, Millville, NJ, USA) in 9 mL of a complete growth medium (RPMI–1640, 10% FBS, 1% MEM non-essential amino acid solution, 1% penicillin–streptomycin, 1% HEPES, 1% sodium pyruvate; Sigma Aldrich, Schnelldorf, Germany), and filtered (70 μm mesh; Falcon, Tewksbury, MA, USA). A homogenous suspension was obtained and the centrifuged cell pellets (450× *g*, 10 min, 25 °C) were resuspended in 3 mL of a complete growth medium (Sigma Aldrich, Schnelldorf, Germany). Thus-prepared cells from the spleen and diluted blood samples were gently layered on 3 mL of Histopaque-1077 (Sigma Aldrich, Schnelldorf, Germany) and centrifuged (400× *g*, 30 min, 25 °C). The resultant buffy coat of mononuclear cells was carefully collected into sterile test tubes, rinsed twice with PBS (Sigma Aldrich, Schnelldorf, Germany) containing 1% FBS (Sigma Aldrich, Schnelldorf, Germany), and resuspended in 1 mL of PBS (Sigma Aldrich, Schnelldorf, Germany). 

Bursa of Fabricius samples were cut into 2 mm pieces and digested with a collagenase type IV solution (RPMI-1640, 1% HEPES, 1% Penicillin–Streptomycin, Collagenase Type IV; Sigma Aldrich, Schnelldorf, Germany). After filtration (70 µm mesh; Falcon, Tewksbury, MA, USA), cell suspensions were rinsed twice and centrifuged (450× *g*, 10 min, 20 °C) in the complete growth medium (Sigma Aldrich, Schnelldorf, Germany) to remove residues of the collagenase solution. Next, the cell pellets were resuspended in 2.5 mL of 40% Percoll density gradient (Sigma Aldrich, Schnelldorf, Germany) and gently layered on 2.5 mL of 60% Percoll. After centrifugation (900× *g*, 20 min, 21 °C), the mononuclear cells were collected from the interphase, rinsed twice in PBS with 1% FBS (Sigma Aldrich, Schnelldorf, Germany), and resuspended in 1 mL of PBS (Sigma Aldrich, Schnelldorf, Germany).

Cell concentrations and the percentage of viable cells isolated from peripheral blood, spleen and bursa of Fabricius were determined in a Vi-cell XR analyzer (Beckman Coulter, Brea, CA, USA).

### 2.8. Determination of Anti-PiCV rCP B IgY-SBC in Spleen Samples by ELISPOT

ELISPOT was performed, as described previously [36], with modifications. After membrane activation (70% ethanol, 50 μL/well, 1 min) and 4 PBS (Sigma Aldrich, Schnelldorf, Germany) rinsing cycles (300 µL/well), MultiScreen plates (Merck Millipore, Burlington, MA, USA) were coated with 100 µL/ well of the PBS solution of PiCV rCP (conc. 20 µg/1 mL) and incubated at 4 °C for 24 h. The plates were then rinsed 3 times with PBS-Tween 20 solution (0.05%; Sigma Aldrich, Schnelldorf, Germany) and once with PBS (Sigma Aldrich, Schnelldorf, Germany), then blocked with the RPMI medium containing 20% FBS (Sigma Aldrich, Schnelldorf, Germany) at 37 °C for 1 h. The samples were standardized to 1.5 × 10^5^ cells and deposited directly in the wells of the previously prepared plates. Mononuclear cells from each sample were deposited in triplicate. Next, 100 µL of Iscove’s modified Dulbecos medium (IMDM; Sigma Aldrich, Schnelldorf, Germany) was added to each well. After incubation (39.5 °C, 5% CO_2_, 24 h), the plates were rinsed 3 times with PBS-Tween 20 (Sigma Aldrich, Schnelldorf, Germany), 3 times with PBS (Sigma Aldrich, Schnelldorf, Germany), and incubated overnight (4 °C) with biotinylated rabbit anti-pigeon IgY (Antibodies-online, Atlanta, GA, USA). The plates were then rinsed 4 times with PBS (Sigma Aldrich, Schnelldorf, Germany), and after loosening the bottom of the plate, the membranes were rinsed with PBS (Sigma Aldrich, Schnelldorf, Germany) on the reverse. Next, 100 µL of streptavidin-alkaline phosphatase (S-AP) (Vector Laboratories, Burlingame, CA, USA) dissolved (1:500) in PBS (Sigma Aldrich, Schnelldorf, Germany) were added to each well. After incubation at 25 °C for 1 h, the plates were rinsed again as described above. Enzymatic reaction was performed with an alkaline phosphatase substrate (BCIP/NBT; Vector Laboratories, Burlingame, CA, USA) for 15–25 min and stopped with distilled water. Anti-PiCV rCP B IgY^+^ spot-forming units (SFU) were counted with an Eli.Scan plate scanner and Eli.Analyse software (A-EL-VIS, Hannover, Germany). Data was expressed as the mean absolute number of SFU ± SD per 1 × 10^6^ of mononuclear cells in each group for each day of sampling. 

### 2.9. Monoclonal Antibodies (mabs) against Pigeon CD3^+^, CD4^+^ and CD8^+^ T Lymphocytes

The mabs against pigeon CD3^+^, CD4^+^ and CD8^+^ T lymphocytes are not commercially availble. For this reason they were developed and provided by Dr. Arkadiusz Miazek, M.Sc.Eng. Marta Lisowska and Dr. Andrzej Rapak from Hirszfeld Institute of Immunology and Experimental Therapy (Wrocław, Poland) as an external service (see Acknowledgments for details).

Specificity of the mabs was tested with the flow cytometry method using mononuclear cells from pigeon, chicken, and turkey blood. The monoclonal antibodies entered into reactions only with the pigeon cells (Figure S1).

### 2.10. Extracellular Staining for T CD3^+^, CD4^+^, CD8^+^ And B IgM^+^ Lymphocytes

Before the experiment, the optimal concentration of monoclonal antibodies was evaluated by staining isolated mononuclear cells with serial dilutions (1 to 10 µL per one million cells) of mabs. The optimal concentrations of mabs were 7 µL/ one million cells (anti-CD3 and anti-CD8 antibodies) and 8 µL/ one million cells (anti-CD4 antibodies). The cross-reactivity of goat anti-chicken IgM-FITC polyclonal antibodies (AbD Serotec, Kidlington, UK) and their optimal concentration were evaluated in the previous study [37]. 

Thereafter, half a million mononuclear cells, isolated from each sample, were stained for certain T and B cell subpopulations according to the following scheme. The samples from blood and spleen were divided into four parts and each one was stained with one of the following antibodies: anti-CD3 (FITC), anti-CD4 (FITC), anti-CD8 (FITC) mabs, and anti-IgM (FITC) polyclonal antibodies. Samples obtained from the bursa of Fabricius were divided into two parts and each one was stained with anti-CD3 (FITC) mabs or anti-IgM (FITC) polyclonal antibodies. The staining strategy for flow cytometry is presented in Figure S2. After staining, the samples were incubated in darkness on ice for 30 min. Next, the cells were twice rinsed in PBS (Sigma Aldrich, Schnelldorf, Germany), centrifuged (400× *g*, 10 min), and the resulting pellets were resuspended in 400 µL of PBS (Sigma Aldrich, Schnelldorf, Germany) and analyzed using a FACSCanto II (BD Biosciences, Franklin Lakes, NJ, USA) flow cytometer. Data were acquired in FACSDiva Software 6.1.3. (BD Biosciences, Franklin Lakes, NJ, USA). Cells were analyzed and immunophenotyped in FloJo 7.5.5 (Tree Star, Ashland, OR, USA). Data were expressed as the mean percentage of a certain subpopulation of lymphocytes ± standard deviation in each group for each day of sampling. 

### 2.11. RNA Isolation and qPCR for IFN-γ Gene Expression

The number of mononuclear cells isolated from spleen samples was standardized to 5 × 10^6^ and used for RNA isolation performed with a NucleoSpin RNA kit (Macherey Nagel, Düren, Germany) according to the manufacturer’s protocol. RNA quality was evaluated in a 2100 Bioanalyzer (Agilent, Santa Clara, CA, USA). Concentrations of eluted RNA were measured with a NanoDrop 2000 spectrophotometer (Thermo Fisher Scientific, Waltham, MA, USA), and the samples were stored at −80 °C until further analysis.

Reverse transcription was carried out with a High-Capacity cDNA Reverse Transcription Kit (Life Technologies, Carlsbad, CA, USA) according to the manufacturer’s recommendations. The concentration of RNA was standardized to 0.5 µg per sample. The expression of the gene encoding IFN-γ was determined with the qPCR method described previously [37] using a Power SYBR^®^ Green PCR Master Mix kit (Life Technologies, Carlsbad, CA, USA) and LightCycler 96 (Roche, Basel, Switzerland). The relative expression was calculated using the ΔCq method [38] normalized to efficiency corrections, average qPCR repeats and reference gene coding glyceraldehyde 3-phosphate dehydrogenase (GAPDH) in GenEx v. 6.1.0.757 data analysis software (MultiD, Göteborg, Sweden). 

### 2.12. Statistical Analysis

The significance of differences in all parameters between the investigated groups were analyzed using the U Mann-Whitney non-parametric test with continuity correction for two independent groups using STATISTICA.PL v. 10.0 software (Statsoft, Kraków, Poland). Differences were considered significant at *p* < 0.01 and <0.05.

## 3. Results

### 3.1. Determination of Anti-PiCV rCP IgY with In-House ELISA

**Figure 1:**
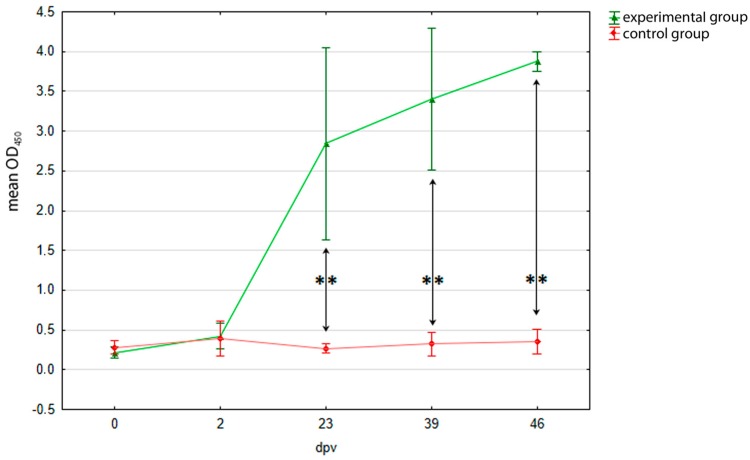
Detection of PiCV rCP–specific IgY in sera of the examined pigeons using in-house ELISA. The pigeons were immunized with PiCV rCP mixed with adjuvant (experimental group, E) or adjuvant only (control group, C) on day 0 and boosted on day 21. Serum samples were collected from birds of both studied groups on days 0, 2, 23, 39, and 46 after first immunization (dpv). The asterisks indicates a statistically significant difference between the investigated groups at *p* < 0.01 in U Mann-Whitney non-parametric test. Error bars represent the standard error of the mean.

Results of the serological examination of pigeons are presented in Figure 1. The initial mean OD_450_ reached 0.21 ± 0.06 and 0.28 ± 0.08 for E and C groups, respectively. In-house ELISA analysis showed that a PiCV rCP antibody response was detected in group E birds on 23 dpv (OD_450_ = 2.84 ± 1.15). The OD_450_ value in group E pigeons gradually increased to the maximum value of 3.87 ± 0.11 on 46 dpv and those birds had significantly higher antibody levels than the control pigeons on 23, 39, and 46 dpv (*p* = 0.00).

### 3.2. Determination of Anti-PiCV rCP B IgY-SBC in Spleen Samples by ELISPOT

**Figure 2:**
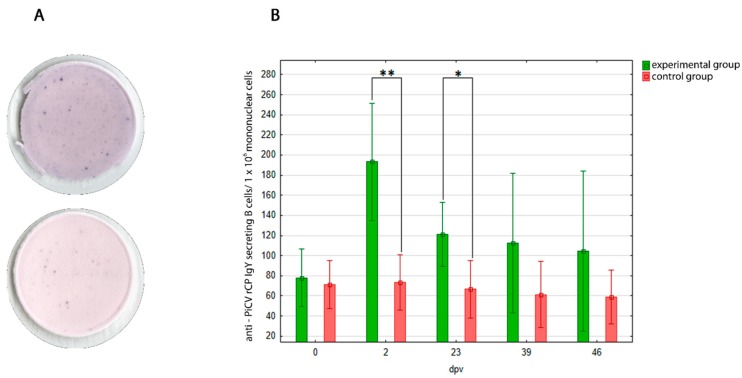
(**A**) Comparison of two representative samples of PiCV rCP immunized pigeons (upper) and control pigeons (lower). Numerous SFU were noted in the samples from pigeons of both groups; nevertheless, in the control group birds the SFU number was constant throughout the experiment, and it was treated as a background. (**B**) The results of ELISPOT for anti-PiCV rCP IgY–SBC in the spleens of the investigated pigeons. The pigeons were immunized with PiCV rCP mixed with adjuvant (experimental group, E) or adjuvant only (control group, C) on day 0 and boosted on day 21. Spleen samples were collected from birds of both studied groups on days 0, 2, 23, 39, and 46 after first immunization (dpv). The asterisks indicates a statistically significant difference between the investigated groups, where * *p* < 0.05, ** *p* < 0.01 in U Mann-Whitney non-parametric test. Error bars represent the standard error of the mean.

Results of the ELISPOT assay are presented in Figure 2. Spot-forming units were noted in the samples obtained from both groups of pigeons, but in the PiCV rCP immunized birds, the number of SFU was higher. The mean number of SFU in the spleen of PiCV rCP immunized pigeons was the highest on 2 dpv (193.33 ± 55.45 per 1 × 10^6^ of mononuclear cells) and successively decreased to the value of 104.44 ± 75.62 per 1 × 10^6^ of mononuclear cells on 46 dpv. The mean number of SFU in the spleens of control pigeons was constant throughout the experiment. The difference in SFU number in spleens between both groups of birds was significant only on two (*p* = 0.00) and 23 (*p* = 0.01) dpv, because of high SD value (39.3–75.1).

### 3.3. Flow Cytometry for T CD3^+^, CD4^+^, CD8^+^ and B IgM^+^ Lymphocytes

Results of extracellular staining for B IgM^+^ lymphocytes in the blood, spleens, and bursa of Fabricius of the examined pigeons are presented in Table 1. The percentage of B IgM^+^ in the blood approximated 7.13 ± 0.92% in pigeons from both groups. In all sampling dates, except the fourth one (39 dpv), the percentage of this lymphocyte subpopulation was negligibly higher in the control than in the PiCV rCP immunized birds, but the differences were not statistically significant. 

In the case of spleen samples, the IgM^+^ percentage did not differ significantly between both investigated groups of pigeons, and reached 13.7 ± 2% on average.

The percentage of B IgM^+^ in the bursa of Fabricius in the control group pigeons was constant and reached 37.3 ± 3% on average. In the pigeons immunized with PiCV rCP, it varied from 32.41 ± 2.64% to 38.63 ± 1.26% throughout the experiment. On 39 dpv, the percentage of B IgM^+^ was significantly lower in E than in C group pigeons (*p* = 0.04).

The dynamics on percentage of selected T lymphocyte subpopulations is presented in Table 2. The percentage of T CD3^+^ lymphocytes in blood was not significantly lower in the PiCV rCP immunized group than in the control pigeons both before and after the immunization, except for 39 dpv. 

In the spleen samples, the percentage of T CD3^+^ lymphocytes subpopulation varied during the whole experiment from 47.78 ± 4.45% to 57.71 ± 2.93% in group E pigeons and from 51.13 ± 1.83% to 61.88 ± 4.25% in group C birds. On 23 dpv, the percentage of this subpopulation of lymphocytes was significantly lower in E than in C group birds (*p* = 0.04). 

In the bursa of Fabricius, the percentage of T CD3^+^ was similar in both investigated groups throughout the experiment and significantly lower on 23 dpv in group E pigeons (*p* = 0.04).

The percentage of T CD4^+^ cells did not differ significantly in blood samples of pigeons from both investigated groups and reached on average 12.91 ± 3.01% and 13.04 ± 3.87% for E and C groups, respectively. However, on two and 23 dpv, the percentage of this subpopulation of lymphocytes was inconsiderably higher in the control group pigeons than in the immunized birds, whereas on 39 dpv, the percentage of this T cell subpopulation was higher in group E birds than in the control ones.

As is presented in Table 2, the percentage of T CD4^+^ lymphocytes in the spleen was higher in the pigeons immunized with PiCV rCP than in the control group birds during almost the entire experiment (21.92 ± 7.18% and 20.05 ± 5.71% on average for E and C groups, respectively), but the difference appeared significant (*p* = 0.04) only on 39 dpv.

The CD8^+^ T cells’ percentage in blood samples varied from 10.3 ± 3.68% to 13.26 ± 2.23% and from 8.76 ± 2.86% to 15.49 ± 4.01% in the pigeons from groups E and C, respectively. The percentage of this subpopulation of T cells was higher in group E pigeons than in the control birds only before immunization, whereas on two and 23 dpv, it was lower in the birds from this group in comparison to the control group birds, but the differences were statistically non-significant. In the other sampling terms, the percentage of T CD8^+^ lymphocytes in blood samples was similar in both investigated groups. 

There were no significant differences between the examined groups in the percentage of T CD8^+^ lymphocytes in the spleen samples. Nevertheless, the percentage of this subpopulation of lymphocytes in the spleens of E group pigeons was non-significantly higher than in the control group before and on 39 and 46 dpv.

### 3.4. qPCR for IFN-γ Gene Expression

**Figure 3:**
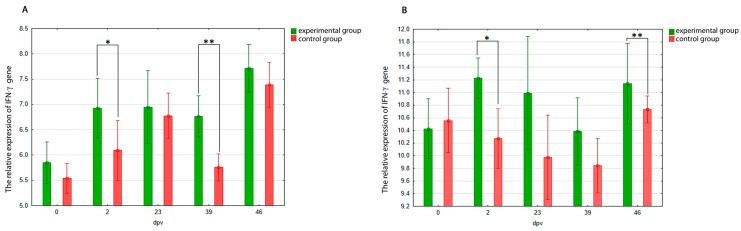
Mean relative expression of the genes encoding IFN-γ in blood (**A**) and splenic (**B**) mononuclear cells of pigeons immunized with PiCV rCP mixed with adjuvant (experimental group, E) or adjuvant only (control group, C) on day 0 and boosted on day 21. Samples were collected from pigeons of both studied groups on days 0, 2, 23, 39, and 46 after first immunization (dpv). The asterisks indicate a statistically significant difference between investigated groups, where * *p* < 0.05, ** *p* < 0.01 in U Mann-Whitney non-parametric test. Error bars represent the standard error of the mean.

In blood samples the expression of gene encoding IFN-γ was higher in group E pigeons than in control group during whole experiment, but only on two and 39 dpv the differences were statistically significant (*p* = 0.01 and *p* = 0.00, respectively) (Figure 3A). The expression of IFN-γ gene in spleen samples was inconsiderably higher in the pigeons immunized with PiCV rCP than in the control group throughout the post immunization period, but only on two and 46 dpv were the differences significant (*p* = 0.01 and *p* = 0.00, respectively) (Figure 3B).

## 4. Discussion

Racing pigeon breeders face a growing problem of diseases with a viral etiology, in particular, infections with PiCV, which is the most frequently diagnosed virus in pigeons [7]. Considering the high prevalence of PiCV in pigeon flocks, the development of an effective vaccine against this virus could be a strategy for the prophylaxis of YPDS, as has been done in the case of swine circovirus [31,32,33]. Currently, gene-engineered vaccines based on the major immunogens like capsid proteins of viruses are under development in both human and veterinary medicine. In recent years, recombinant capsid proteins of various circoviruses like DuCV, PCV2, PBFDV and PiCV have been produced in bacterial, yeast, and baculovirus systems [10,26,29,30,31,32,33,34,39,40,41]. Little is known, however, about the immunogenicity of avian circoviruses’ recombinant capsid proteins. For the above reasons, an experiment was designed to evaluate the immunogenicity of PiCV rCP in pigeons. While SPF pigeons are unobtainable, all birds used in this study originated from a flock with no YPDS history, and all pigeons were not positive for PiCV genetic material nor for antibodies against this virus. 

To check the immunogenicity of PiCV rCP, both the humoral and the cell-mediated immune mechanisms were investigated. Antibody response to a defined antigen, such as purified protein, is more useful in studies of the humoral immune system than the measurement of antibody response to a complex antigen, like that found in pathogens, which is an advantage of the present study. 

The M class antibodies are produced first and constitute the largest group of immunoglobulins [42]. In the present study, the percentage of IgM^+^ B cells was similar in both investigated groups throughout the experiment in the samples from each organ except the bursa of Fabricius, where this subpopulation of B lymphocytes was smaller in the immunized birds on 39 dpv. However, the percentage of IgM^+^ B cells in the PiCV rCP immunized pigeons was more diverse throughout the experiment than in the control birds, which is clearly visible in the case of the bursa of Fabricius and blood (Table 1). Those differences seem to have no relationship with the experimental factor, which is immunization with PiCV rCP, because the fortuity of differences could result from inter-individual differences between the examined pigeons. In light of the above, we could conclude that immunization with PiCV rCP is not sufficient to stimulate remarkable changes in the subpopulation of these B lymphocytes. 

The IgY, which are homologs to mammalian IgG, are the predominant form of antibodies in avian sera and are produced after IgM in the primary antibody response and as the main antibodies produced in the secondary response. In our study, results of in-house ELISA showed that the level of anti-PiCV rCP IgY increased successively in the immunized pigeons, which was indicated by an increasing OD_450_ value. The seroconversion was detectable in the immunized birds on 23 dpv, where OD_450_ value reached 2.84 ± 1.15. The OD_450_ value peaked to 3.87 ± 0.11 on 46 dpv in group E pigeons, while no OD_450_ changes were detected in the control birds (Figure 1). The differentiation between samples influencing the SD value gradually decreased after 23 dpv, which shows a correct immune response to the antigen. The obtained results are in concordance with those reported for parrots and swine, where high levels of antibodies were detected in the animals immunized with circovirus recombinant capsid proteins two weeks after the first vaccination [26,31,33,34,43]. 

The B-cell ELISPOT technique used in this study calculated the mean absolute number of the anti-PiCV rCP IgY–SBC. To obtain reliable results, the number of cells deposited into ELISPOT plate wells was equal; each sample was deposited in triplicate and average number of SFU was calculated for each sample. As it appears from our study, subsequent immunizations of pigeons with PiCV rCP contributed to the successive reduction in the number of anti-PiCV rCP IgY-SBC since 2 dpv. Nevertheless, on each sampling date, the SFU number was higher in the experimental group than in the control birds, in which the constant SFU number was treated as a background. Due to the high variability between the samples, the differences observed between the investigated groups of pigeons were statistically significant only on two and 23 dpv (Figure 2B). The decreasing number of anti-PiCV rCP IgY-SBC may be explained as resulting from the successive extinguishment of reaction force of this component of humoral immunity resulting from repeated exposure to the antigen with relatively low immunogenicity. Comparison of the results of in-house ELISA and ELISPOT reveals an opposite dependency between levels of anti-PiCV rCP IgY in pigeon sera and anti-PiCV rCP IgY-SBC in the spleen. The lifespan of plasma cells (IgY-SBC) is lower than the half-life period of specific antibodies, and only a small population of antibody-secreting cells appears later as memory cells [44]. Because IgY are secreted by each generation of plasma cells and are detectable after plasmocyte death, their number increases even if the number of IgY-SBC decreases. This dependency was observed not only in our study, but also in the research conducted with the infectious bronchitis virus (IBV). Pei et al. (2005) showed that the number of anti-IBV IgY-SBC increased in chicken spleens on days three and seven post inoculation (dpi), and then the number of those cells sharply decreased by more than 20-fold by 14 dpi. The anti-IBV IgY-SBC number was then low and stable until week 10 post inoculation (pi). Whereas specific IgY antibodies against IBV were detected at low levels seven days later than anti-IBV IgY-SBC, their level increased successively up to two weeks pi and they were still present at a high level up to 10 weeks pi [44]. The kinetics of anti-PiCV rCP-SBC and antibody response to PiCV rCP in the present study is very similar to this described above.

T lymphocytes can be divided into subpopulations based on the presence of cell surface receptors, and can be measured in peripheral blood and lymphatic organs by monoclonal antibodies and flow cytometry techniques. Determination of the percentage of selected T cell subpopulations could provide interesting information about the immune response of the organism to infection or vaccination; such analyses are successfully performed in various poultry species with commercial antibodies [45,46,47]. Unfortunately, no commercially available monoclonal antibodies are dedicated for pigeons. Our previous investigation using anti-chicken monoclonal antibodies for staining pigeon T cells was saddled with high methodological error due to very limited cross reactivity between the used antibodies and pigeon T cells [48]. For this reason, a protocol was developed for producing monoclonal antibodies against pigeon T cells’ surface receptors. The purified antibodies were checked for cross-reactivity with mononuclear cells isolated from chicken and turkey, and no-cross reactions were detected. For the first time, we show flow cytometry analyses performed using anti-pigeon T cell receptor monoclonal antibodies. In the presented study, no significant differences were noted in the percentage of T CD3^+^, CD4^+^ and CD8^+^ cells in the post-immunization period between the investigated groups, except CD3^+^ T cells in the bursa of Fabricius on 23 dpv and CD4^+^ T cells in the spleen on 39 dpv. Nevertheless, the average percentage of T CD3^+^ lymphocytes in all examined organs was a bit lower in the immunized birds than in the control group, whereas that of CD4^+^ T lymphocytes was usually higher in the immunized birds and that of CD8^+^ T cells was at a similar level. Randomness of the above differences and high variability in values of the same parameter between sampling dates in particular groups suggest that they resulted from factors other than immunization, probably from inter-individual differences and increasing age of the birds. The diversity of certain T cell populations in relation to the age of birds is often noted in avian species [49,50]. The obtained results suggest that immunization with PiCV rCP has no influence on the percentage of the examined subpopulation of T cells; this conclusion is convergent with the results obtained by other authors who were also unable to detect clear differences in T cell subpopulations between unvaccinated piglets and those immunized with porcine circovirus sub-unit vaccine [51]. In the case of swine, changes were observed over time in the post-immunization period in various T cell subpopulations in both immunized groups were attributed to the physiological development of immune competence in growing pigs [51]. In view of the presented results and literature data, it seems immunization with PiCV rCP is not sufficient to stimulate changes in certain T lymphocyte subpopulations.

The immune reaction to a viral infection and vaccination also leads to enhanced synthesis of interferons, especially IFN-γ, which plays a significant role in both immediate and long-term immune responses. IFN-γ is secreted mainly by T CD4^+^ and CD8^+^ lymphocytes and to a lesser extent by B cells, NK cells, and NKT cells [52]. Increasing research evidence has suggested that the specific cellular response—including IFN-γ secretion—is involved in the protection against PCV2-SD [32,34,51,53]. In this study, we learned that immunization with PiCV rCP stimulated IFN-γ gene expression. It is noteworthy that the expression of this gene was higher in the immunized group during the whole post-immunization period, but for the reason of high standard deviation the differences were statistically significant only on two and 39 dpv in blood and on two and 46 dpv in the spleen. Our results are in concordance with the investigation conducted on pigs, where a stimulatory effect of PCV2 rCP on IFN-γ production was observed within 42 days after vaccination [34,51]. The increased interferon production for a relatively long (six-week) period after the first immunization could result from the fact that the antigen was mixed with the adjuvant in both our study and the cited study. An earlier investigation conducted on pigs showed that the addition of the adjuvants to PCV2 rCP was necessary to enhance the immune response in vaccinated animals, because recombinant Cap protein of this virus alone induces low IFN-γ response [54]. The stimulatory effect of PiCV rCP on this gene expression could be reflected in practice, because, in the case of swine circovirus, the humoral response alone is not enough to provide complete protection for pigs against PCV2-SD, and cell-mediated immunity including IFN-γ response promotes the efficacy of the vaccines against this disease [31,32]. No similar data exists concerning PiCV and pigeons, but considering results obtained in this study and findings reported in the available literature as well as similarities between YPDS and PCV2-SD, an analogous mechanism could be involved in the immune response against PiCV.

## 5. Conclusions

Under conditions of this basic research, pigeon immunization with PiCV rCP induced both humoral and cell-mediated immunity, which was manifested by a significant increase in the number of IgY from 23 days post first immunization, by an increase in the number of anti-PiCV rCP IgY-SBC since 2 dpv, and by stimulated expression of IFN-γ in blood and splenic mononuclear cells since two dpv. The high levels of specific antibodies and IFN-γ play an important role in the prevention and reduction of infection or clinical signs in the case of swine circovirus [31,32,43,51] and, by analogy, similar or the same mechanism could be involved in the immunity against pigeon circovirus. In the light of the obtained results, it may be concluded that pigeon circovirus recombinant capsid protein could be an antigen candidate in sub-unit vaccines against PiCV infections in pigeons. This vaccine could protect pigeons against such infections, and possibly, lower the prevalence of YPDS. However, considering the high prevalence of PiCV, the subclinically infected pigeons will be vaccinated, if the vaccine is developed. For the above reasons, more basic research should be performed to evaluate the immune response to PiCV rCP in both uninfected and asymptomatically infected pigeons. In turn, application research with experimental challenge with PiCV is also needed to evaluate the protectivity of the vaccine based on PiCV rCP.

## Figures and Tables

**Table 1:** The percentage of B IgM^+^ lymphocytes in the blood, spleen, and bursa of Fabricius samples collected from the examined pigeons. Abbreviations: dpv—days post first immunization, M—mean, SD—standard deviation, E-experimental group (immunized with PiCV rCP mixed with adjuvant), C—control group (immunized with adjuvant only). The values in the same column with different superscripts (^a,b^) differ significantly at *p* < 0.05 in U Mann-Whitney non-parametric test.

Group		B IgM^+^ (%)					B IgM^+^ (%)					B IgM^+^ (%)				
		Blood					Spleen					Bursa of Fabricius				
		dpv					dpv					dpv				
		0	2	23	39	46	0	2	23	39	46	0	2	23	39	46
E	M	6.18	7.09	6.82	8.84	6.51	13.53	12.91	13.76	14.63	13.66	36.26	34.85	38.48	32.41 ^b^	38.63
	SD	0.94	0.63	0.79	1.75	1.23	0.91	1.46	2.14	2.33	1.22	2.6	2.98	2.75	2.64	1.26
C	M	6.5	7.26	7.66	7.25	7.28	12.95	12.76	14.28	14.3	14.08	36.3	36.83	37.86	36.86 ^a^	38.51
	SD	0.69	0.53	0.59	1.33	0.78	1.51	0.96	3.12	2.55	1.65	3.14	2.77	2.69	3.85	3.35

**Table 2:** The percentage of T CD3^+^, CD4^+^ and CD8^+^ lymphocytes in the blood, spleen and bursa of Fabricius samples collected from the examined pigeons. Abbreviations: dpv—days post first immunization, M—mean, SD—standard deviation, nd—not determined, E—experimental group (immunized with PiCV rCP mixed with adjuvant), C—control group (immunized with adjuvant only. The values in the same column with different superscripts (a, b) differ significantly at *p* < 0.05 in U Mann-Whitney non-parametric test.

Group		CD3 ^+^ (%)					CD4^+^ (%)					CD8^+^ (%)				
		dpv					dpv					dpv				
		0	2	23	39	46	0	2	23	39	46	0	2	23	39	46
							blood									
E	M	32.38	23.23	28.43	37.6	30.98	10.71	11.14	11.01	13.74	13.83	10.3	10.52	11.54	11.25	13.26
	SD	3.76	5.71	4.55	4.56	4.98	3.06	1.98	3.36	3.07	2.62	3.68	3.32	2.94	3.99	2.23
C	M	39.58	28.88	34.76	35.23	33.63	10.03	14.37	15.8	11.41	13.61	8.76	13.19	15.49	11.12	13.34
	SD	8.28	6.27	4.21	3.43	5.64	3.17	4.15	4.03	2.54	3.34	2.86	4.68	4.01	3.32	2.98
							spleen									
E	M	50.00	52.05	47.78^b^	54.93	57.71	29.26	26.13	17.3	16.18^a^	20.75	28.48	26.08	20.06	19.93	20.73
	SD	4.52	3.94	4.45	6.40	2.93	7.42	6.52	1.16	4.73	5.04	5.19	5.38	2.76	4.32	2.58
C	M	53.73	51.13	53.68^a^	61.88	53.8	26.08	24.01	19.00	12.1^b^	19.08	26.1	27.5	24.46	18.75	19.07
	SD	6.38	1.83	4.28	4.25	3.32	3.82	4.99	2.46	2.16	4.39	4.13	3.95	4.87	4.08	4.34
bursa of Fabricius																
E	M	5.05	5.05	5.48 ^b^	4.76	6.43	nd					nd				
	SD	0.73	0.79	0.35	1.56	1.90										
C	M	5.99	5.14	7.34 ^a^	6.54	6.51										
	SD	1.02	0.89	1.76	1.2	0.46										

## Supplementary Materials

The following are available online at http://www.mdpi.com/1999-4915/10/11/596/s1, Figure S1: Cross-reactivity evaluation of the obtained monoclonal antibodies against pigeon T cell surface receptors. The cross-reactivity was evaluated with the cells isolated from blood of chicken (**A**), turkey (**B**), and pigeon (**C**). There was no cross-reaction with chicken and turkey mononuclear cells, Figure S2: The strategy for extracellular staining for CD3^+^, CD4^+^ and CD8^+^ T and IgM^+^ B cells in various samples of the examined pigeons. The lymphocyte gate is marked with blue, whereas particular subpopulations of lymphocytes are marked with the following colors: orange-T CD3^+^, light green–T CD4^+^, pink–T CD8^+^, and dark green–B IgM^+^. (**A**) A representative blood sample. (**B**) A representative spleen sample. (**C**) A representative bursa of Fabricius sample. Abbreviations: FSC-A: forward scatter A, SSC-A: side scatter A, Table S1: Experimental design.
