# Supplementary material for: Immunogenicity of Pigeon Circovirus Recombinant Capsid Protein in Pigeons
Source: Viruses. 2018 Oct 31;10(11):596. doi: 10.3390/v10110596 (PMC6265742; doi:10.3390/v10110596)
Supplement: Supplementary file 1 [file viruses-10-00596-s001.zip › Table S1.pdf]

Table S1. Experimental design.

<table>
<tr><th rowspan="2">Group</th><th colspan="8">Day of experiment / day post first vaccination</th></tr>
<tr><th>1-13</th><th>14/0</th><th>14/0</th><th>16/2</th><th>35/21</th><th>37/23</th><th>53/39</th><th>60/46</th></tr>
<tr><td>E</td><td rowspan="2">Adaptation to new conditions</td><td rowspan="2">ELISA
ELISPOT
Flow cytometry
qPCR</td><td>rCP PiCV 20μg/ bird with adjuvant</td><td rowspan="2">ELISA
ELISPOT
Flow cytometry
qPCR</td><td>rCP PiCV 20μg/ bird with adjuvant</td><td colspan="3" rowspan="2">ELISA
ELISPOT
Flow cytometry
qPCR</td></tr>
<tr><td>C</td><td>adjuvant</td><td>adjuvant</td></tr>
</table>
