# Supplementary figures and images for: Immunogenicity of Pigeon Circovirus Recombinant Capsid Protein in Pigeons
Source: Viruses. 2018 Oct 31;10(11):596. doi: 10.3390/v10110596 (PMC6265742; doi:10.3390/v10110596)

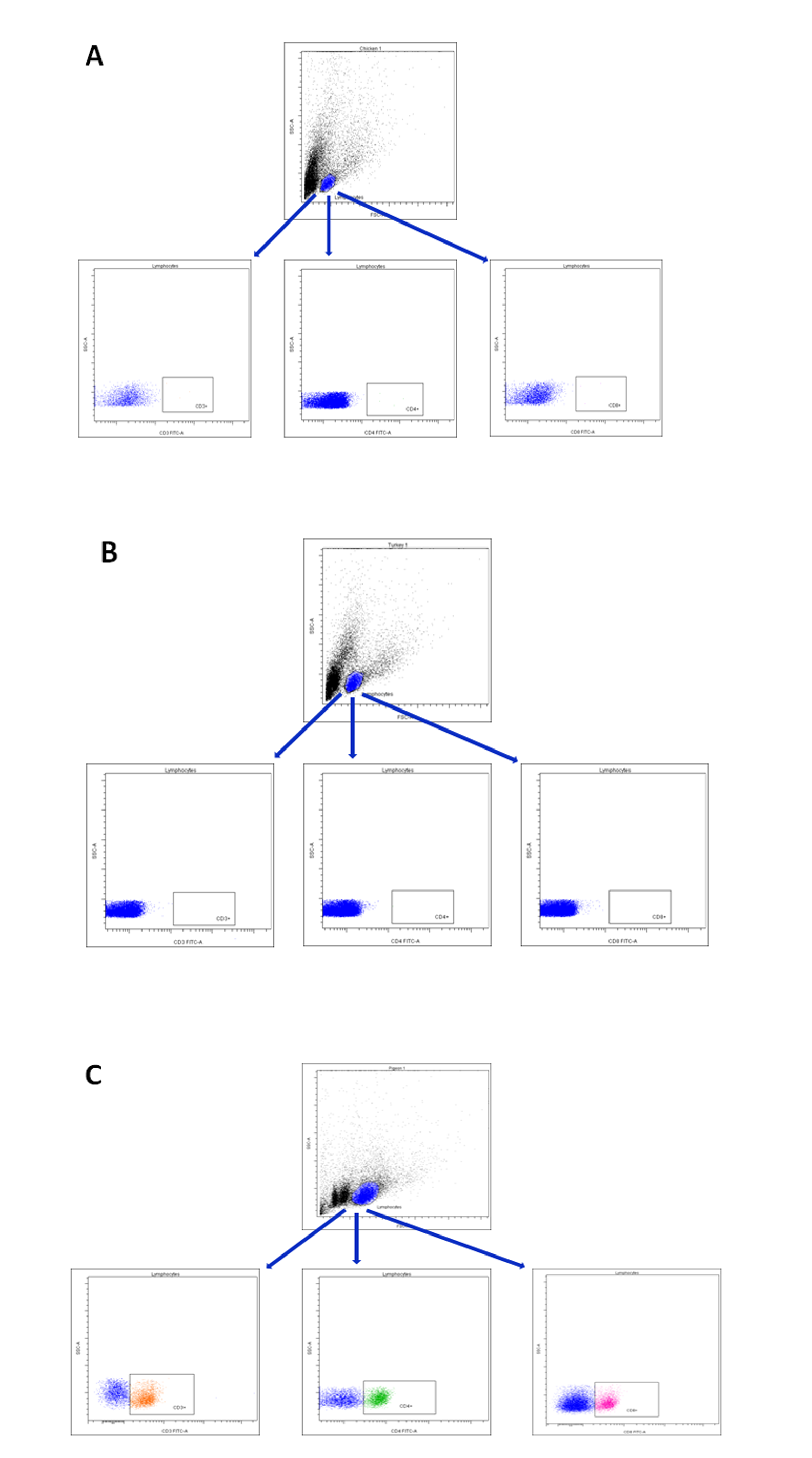

Supplement: Supplementary file 1 [file viruses-10-00596-s001.zip › Figure S1.tif]

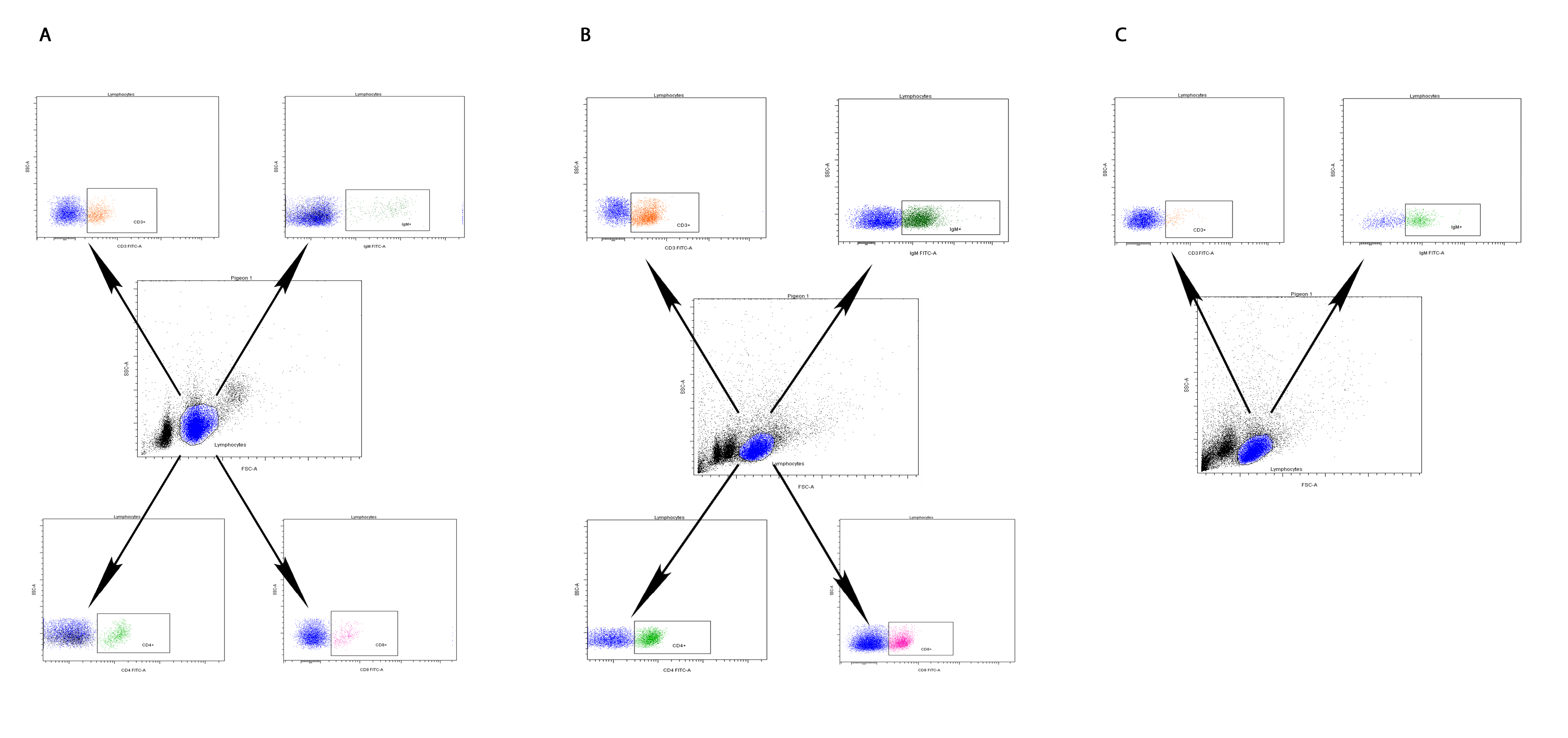

Supplement: Supplementary file 1 [file viruses-10-00596-s001.zip › Figure S2.tif]
